# Supplementary material for: Metabolomic profiling of microbial disease etiology in community-acquired pneumonia
Source: PLoS One. 2021 Jun 4;16(6):e0252378. doi: 10.1371/journal.pone.0252378 (PMC8177549; doi:10.1371/journal.pone.0252378)
Supplement: S1 Method — (DOCX) [file pone.0252378.s001.docx]

**S1 Method: Details on metabolomic sample analysis**

**Batch design**: Aliquoted samples were run in a randomized fashion in several batches together with quality control (QC) samples (every 10 samples), sample replicates (every 7 samples), internal standards (ISTDs), blanks, and calibration lines.

**Quality control**: Blank samples were used to determine the blank effect. Replicate samples were used to check the instrument for repeatability. In-house developed algorithms were applied using the pooled QC samples to compensate for shifts in the sensitivity of the mass spectrometer over the batches.

**Reported results**: After quality control correction the metabolites that complied with the acceptance criteria of a relative standard deviation of the quality control samples (RSDqc) <15% were reported. The data was reported as relative response ratio (analyte signal area / ISTD area; unit free) of the metabolites after QC correction. Metabolites that did not comply with the acceptance criteria of the quality control, but have been included in the results present RSDs up to 30% and should be handled with caution.

**Amine profiling**: Amine profiling was performed according to the validated amine profiling analytical platform with minor optimization [28]. The amine platform covers amino acids and biogenic amines employing an Accq-Tag derivatization strategy adapted from the protocol supplied by Waters. 5,0 µL sample was spiked with an internal standard solution**.** Protein precipitation was performed by addition of MeOH and the sample was dried in a speedvac. The residue was reconstituted in borate buffer (pH 8.5) with AQC reagent. The prepared samples were transferred to autosampler vials and placed in an autosampler tray. The vials were cooled at 4^o^C upon injection. 1,0 µL prepared sample was injected in a UPLC-MS/MS system. Chromatographic separation was achieved by an Agilent 1290 Infinity II LC System on an Accq-Tag Ultra column (Waters) with a flow of 0.7 mL/min over an 11 min gradient. The UPLC was coupled to electrospray ionization on a triple quadrupole mass spectrometer (AB SCIEX Qtrap 6500). Analytes were detected in the positive ion mode and monitored in Multiple Reaction Monitoring (MRM) using nominal mass resolution. Acquired data was evaluated using MultiQuant Software for Quantitative Analysis (AB SCIEX, Version 3.0.2), by the integration of assigned MRM peaks and normalization using proper internal standards. For analysis of amino acids, their 13C15N-labeled analogs were used. For other amines, the closest-eluting internal standard was employed. After quality control correction 48 amines complied with the acceptance criteria of RSDqc <15%. Additionally, 7 amines presented an RSDqc between 15 and 30%. They are included in the results but these compounds should be considered with caution.

**Acylcarnitine profiling**: The acylcarnitine platform covers acylcarnitines as well as trimethylamine-N-oxide, choline, betaine, deoxycarnitine, and carnitine. 10 μL sample was spiked with an internal standard solution. Protein precipitation was performed by addition of MeOH. The supernatant was transferred to an autosampler vial and placed into an autosampler. The vials were cooled at 10°C upon injection. 1.0 μL of the prepared sample was injected into a triple quadrupole mass spectrometer. Chromatographic separation was achieved by UPLC (Agilent 1290, San Jose, CA, USA) on an Accq-Tag Ultra column (Waters) with a flow of 0.7 mL/min over an 11 min gradient. The UPLC was coupled to electrospray ionization on a triple quadrupole mass spectrometer (Agilent 6460, San Jose, CA, USA). Analytes were detected in the positive ion mode and monitored in Multiple Reaction Monitoring (MRM) using nominal mass resolution. Acquired data was evaluated using Agilent MassHunter Quantitative Analysis software (Agilent, Version B.05.01), by integration of assigned MRM peaks and normalization using proper internal standards. The closest-eluting internal standard was employed. After quality control correction 24 acylcarnitines complied with the acceptance criteria of RSDqc <15%. Additionally, 4 acylcarnitines presented an RSDqc between 15 and 30%. They are included in the results but these compounds should be considered with caution.

**Organic acid profiling**: The organic acid platform covers 28 organic acids. 50 μL sample was spiked with an internal standard solution. Protein precipitation was performed by addition of MeOH. After centrifugation, the supernatant was transferred and the sample was dried using a speedvac. Then, two-step derivatization procedures were performed on-line: oximation using methoxyamine hydrochloride (MeOX, 15 mg/mL in pyridine) as the first reaction and silylation using N-Methyl-N-(trimethylsilyl)- trifluoroacetamide (MSTFA) as the second reaction. 1 μL of each sample was directly after its derivatization injected on GC-MS. Gas chromatography was performed on an Agilent Technologies 7890A equipped with an Agilent Technologies mass selective detector (MSD 5975C) and MultiPurpose Sampler (MPS, MXY016-02A, GERSTEL). Chromatographic separations were performed on an HP-5MS UI (5% Phenyl Methyl Silox), 30 m × 0.25 m ID column with a film thickness of 25 μm, using helium as the carrier gas at a flow rate of 1,7 mL/min. A single-quadrupole mass spectrometer with electron impact ionization (EI, 70 eV) was used. The mass spectrometer was operated in SCAN mode mass range 50-500. Acquired data was evaluated using Agilent MassHunter Quantitative Analysis software (Agilent, Version B.05.01). After quality control correction and considering blank effects, 9 organic acid compounds complied with the acceptance criteria RSDqc <15% and blank effect <20%. 4 organic acids reported an RSDqc between 15 and 30% and should be considered with caution**.**

**Negative lipid profiling**: The negative lipid platform is a semi-target methodology for the identification of 30 fatty acids. 50 μL sample was spiked with 50 µL of an internal standard solution. Protein precipitation was performed by addition of 550 µL MeOH. After centrifugation, 600 µL supernatant was transferred and the sample was dried using a speedvac. The residue was reconstituted in 300 µL of isopropanol with 0,1% formic acid. The prepared samples were transferred to autosampler vials and placed in an autosampler tray. 8,0 μL of the prepared sample was injected into an LC-MS. The analysis was performed on an ACQUITY UPLC™ (Waters, the Netherlands) coupled to a high-resolution mass spectrometer with a Synapt G2 Q-TOF system (Waters, the Netherlands) using reference lock mass correction. Lipids were detected in full scan in the negative ion mode. Chromatographic separation was achieved using an HSS T3 column (1.8 μm, 2.1 * 100 mm) with a flow of 0.4 mL/min over a 16-minute gradient. Acquired data was preprocessed using Targetlynx software (Masslynx, V4.1, SCN916). After quality control correction, 10 compounds complied with the acceptance criteria RSDqc <15%. 6 compounds reported an RSDqc between 15 and 30% and should be considered with caution**.**

**Positive lipid profiling**: The positive lipid platform covers 185 compounds including triglycerides (TGs, n=85) and non-triglycerides (non-TGs, n=100). 10 μL preprocessed sample was spiked with 1000 μL IPA containing internal standards and vortexed for 30 sec. Prepared samples were transferred to autosampler vials for LC-MS analysis. In total 2.5 μL prepared sample was injected for analysis. Chromatographic separation was achieved on an ACQUITY UPLC™ (Waters, Ettenleur, the Netherlands) with an HSS T3 column (1.8 μm, 2.1 * 100 mm) with a flow of 0.4 mL/min over a 16 min gradient. The lipid analysis is performed on a UPLC-ESI-Q-TOF (Agilent 6530, Jose, CA, USA) high-resolution mass spectrometer using reference mass correction. Lipids were detected in full scan in the positive ion mode. The raw data were preprocessed using Agilent MassHunter Quantitative Analysis software (Agilent, Version B.04.00). After quality control correction, 56 TGs and 39 non-TGs compounds complied with the acceptance criteria RSDqc <15% and blank effect <40 %. 1 TG and 53 non-TGs reported an RSDqc between 15 and 30% and should be considered with caution.

**Signaling lipid profiling**: The signaling lipids platform covers various isoprostane classes together with their respective prostaglandin isomers from different poly unsaturated fatty acids (PUFA), including n-6 and n-3 PUFAs such as dihomo-γ-linoleic acid (DGLA) and arachidonic acid (both n-6) and eicosapentaenoic acid (EPA) and docosahexaenoic acid (DHA) (both n-3). Also included in this platform are endocannabinoids, bile acids, and signaling lipids from the sphingosine and sphinganine classes and their phosphorylated forms, as well as three classes of lysophosphatidic acids. The three lysophosphatidic acid classes include lysophosphatidic acids (LPAs), lysophosphatidylglycerol (LPG), lysophosphatidylinositol (LPI), lysophosphatidyserine (LPS), lysophosphatidylethanolamines (LPE), cyclic-phosphatidic acids(cLPA), and fatty acid all ranging from C14 to C22 chain length species. The signaling and peroxidized lipids platform is divided into two chromatographic methods: low and high pH. In the low pH method, isoprostanes, prostaglandins, nitro-fatty acids, lyso-sphingolipids, endocannabinoids, and bile acids are analyzed. The high pH method covers lyso-sphingolipids, lysophosphatidic acids, lysophosphatidylglycerol, lysophosphatidylinositol, lysophosphatidyserine, lysophosphatidylethanolamines, cyclic-phosphatidic acids, and fatty acid. Each sample was spiked with antioxidant and internal standard solution. The extraction of the compounds is performed via liquid-liquid extraction (LLE) with butanol and methyl tert-butyl ether (MTBE). After collection, the organic phase is concentrated by first drying followed by reconstituted in a smaller volume. After reconstitution, the extract is transferred into amber autosampler vials and used for high and low pH injection. A Shimadzu system, formed by three high-pressure pumps (LC-30AD), a controller (CBM-20Alite), an autosampler (SIL-30AC), and an oven (CTO-30A) from Shimadzu Benelux, was coupled online with an LCMS-8050 triple quadrupole mass spectrometer (Shimadzu) for high pH measurements. An LCMS-8060 triple quadrupole mass spectrometer (Shimadzu) was coupled to the Shimadzu system for low pH measurements. Both systems were operated using LabSolutions data acquisition software (Version 5.89, Shimadzu). The samples were analyzed by UPLC-MS/MS. An Acquity UPLC BEH C18 column (Waters) was used to measure the samples in the low pH method. For the high pH method, a Kinetex EVO column by Phenomenex was used. The triple quadrupole mass spectrometer was used in polarity switching mode and all analytes were monitored in dynamic Multiple Reaction Monitoring (dMRM). The acquired data was evaluated using LabSolutions Insight software (Version 3.1 SP1, Shimadzu), by integration of assigned MRM peaks and normalization using accordingly selected internal standards. When available, a deuterated version of the target compound was used as an internal standard. For the other compounds, the closest-eluting internal standard was employed. For low pH mode, after quality control correction, 46 metabolites complied with the acceptance criteria of RSDqc <15% and blank effect <40%. 6 compounds reported an RSDqc between 15 and 30% and should be considered with caution. For high pH mode, after quality control correction, 43 metabolites complied with the acceptance criteria of RSDqc <15% and blank effect <40%. Additionally, 18 compounds reported an RSDqc between 15 and 30% and should be considered with caution.
