# Supplementary material for: Metabolomic profiling of microbial disease etiology in community-acquired pneumonia
Source: PLoS One. 2021 Jun 4;16(6):e0252378. doi: 10.1371/journal.pone.0252378 (PMC8177549; doi:10.1371/journal.pone.0252378)
Supplement: S1 Fig — (A) Shows all α and λ values tested in inner CV against mean BER of the inner CV. (B) A plot of the optimal α and λ combinations chosen in the inner CV against their BER in the outer CV shows a variety of favorable α and λ concentrations. (C) A plot of the number of variables selected in the elastic net model in outer CV shows that with increasing alpha, the number of variables decreases as is expected in an elastic net model. The data shown in the Fig is a result of the comparison Atypical–(S. pneumoniae + viral). (DOCX) [file pone.0252378.s002.docx]

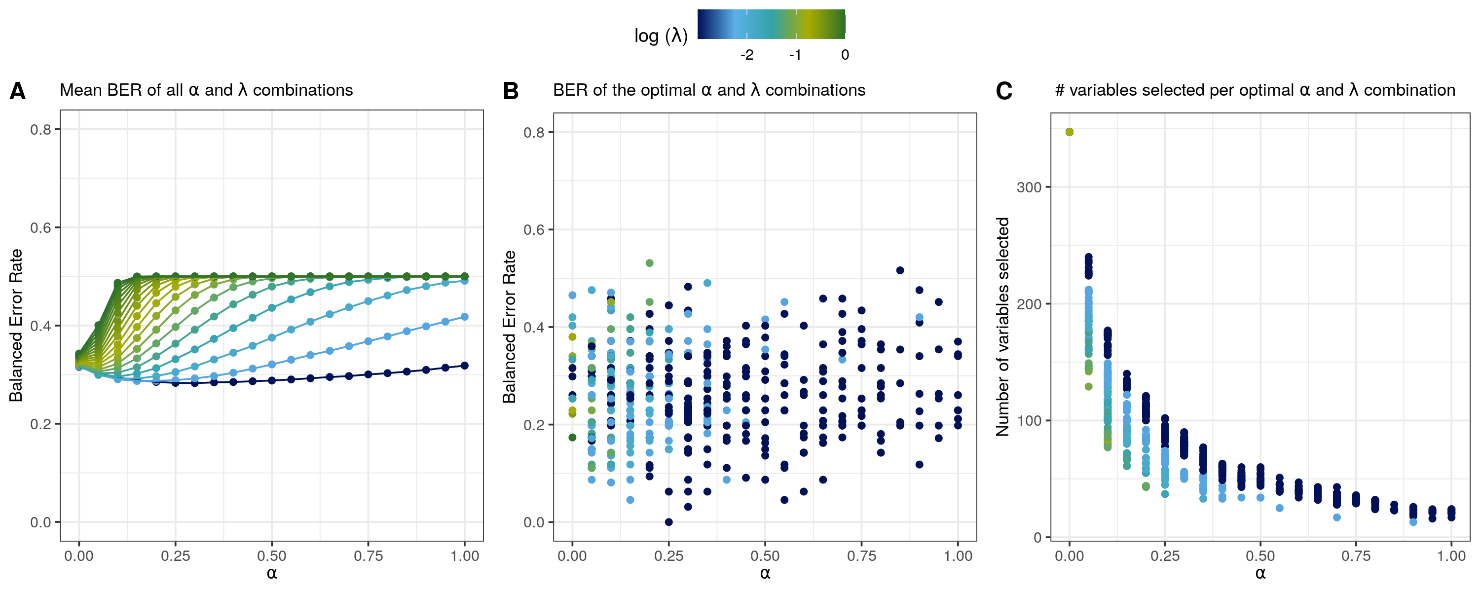


**S1 Fig. Optimization of α and λ in the inner cross-validation (CV) to reach a minimal balanced error rate (BER) in the outer CV.** (A) Shows all α and λ values tested in inner CV against mean BER of the inner CV. (B) A plot of the optimal α and λ combinations chosen in the inner CV against their BER in the outer CV shows a variety of favorable α and λ concentrations. (C) A plot of the number of variables selected in the elastic net model in outer CV shows that with increasing alpha, the number of variables decreases as is expected in an elastic net model. The data shown in the Fig is a result of the comparison Atypical – (S. pneumoniae + viral).
