# Supplementary figures and images for: Metabolomic profiling of microbial disease etiology in community-acquired pneumonia
Source: PLoS One. 2021 Jun 4;16(6):e0252378. doi: 10.1371/journal.pone.0252378 (PMC8177549; doi:10.1371/journal.pone.0252378)

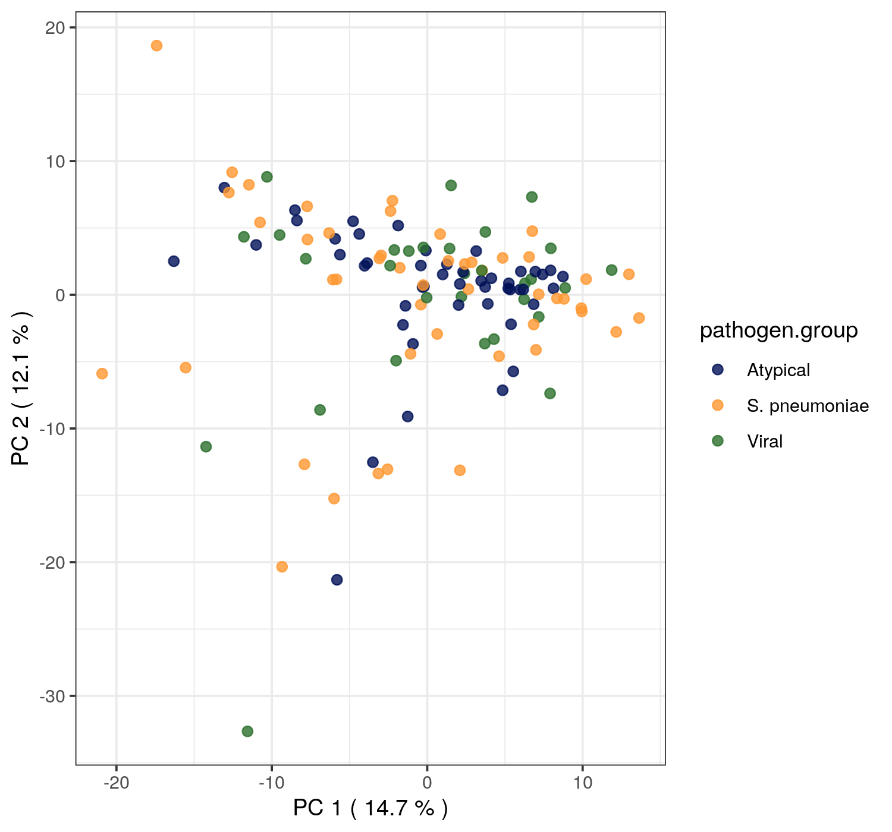


**S2 Fig. Unsupervised principal component analysis (PCA) plot of all pathogen groups.**

Supplement: S2 Fig — (DOCX) [file pone.0252378.s003.docx]
