# Supplementary material for: Metabolomic profiling of microbial disease etiology in community-acquired pneumonia
Source: PLoS One. 2021 Jun 4;16(6):e0252378. doi: 10.1371/journal.pone.0252378 (PMC8177549; doi:10.1371/journal.pone.0252378)
Supplement: S3 Fig — (A) Boxplot of BER per number of variables selected shows no clear relation between the number of variables selected and model performance. (B) Histogram of the number of variables selected shows that a model with all metabolites included is favored, followed by models including 34, 49, 82, 24, or 45 metabolites. Both Figs contain the data of all folds and repeats (n = 500) for the comparison between atypical versus S. pneumoniae and viral infections. (DOCX) [file pone.0252378.s004.docx]

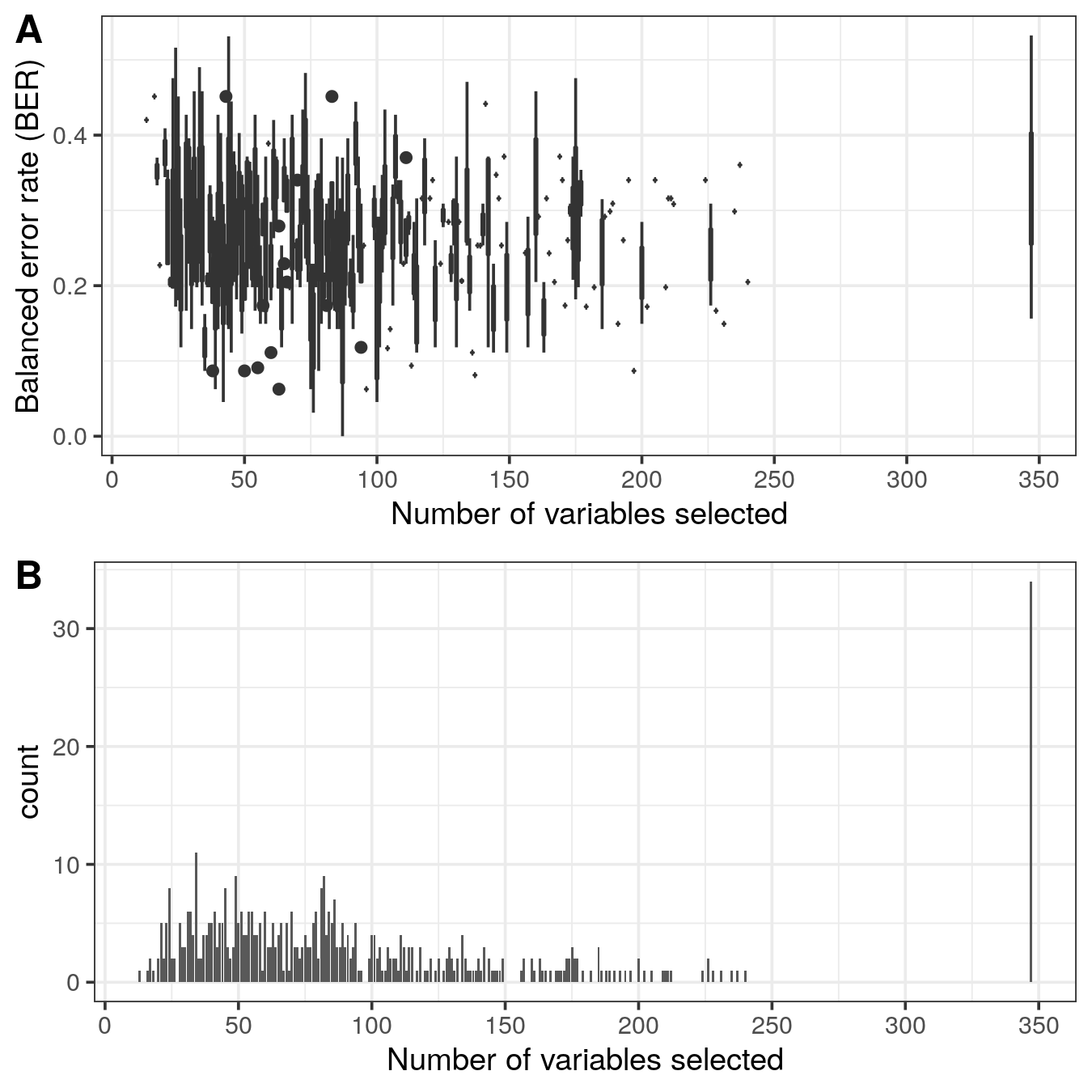


**S3 Fig. (A) Boxplot of BER per number of variables selected shows no clear relation between the number of variables selected and model performance. (B) Histogram of the number of variables selected shows that a model with all metabolites included is favored, followed by models including 34, 49, 82, 24, or 45 metabolites.** Both Figs contain the data of all folds and repeats (n=500) for the comparison between atypical versus S. pneumoniae and viral infections.
