# Supplementary material for: Metabolomic profiling of microbial disease etiology in community-acquired pneumonia
Source: PLoS One. 2021 Jun 4;16(6):e0252378. doi: 10.1371/journal.pone.0252378 (PMC8177549; doi:10.1371/journal.pone.0252378)
Supplement: S4 Fig — (DOCX) [file pone.0252378.s005.docx]

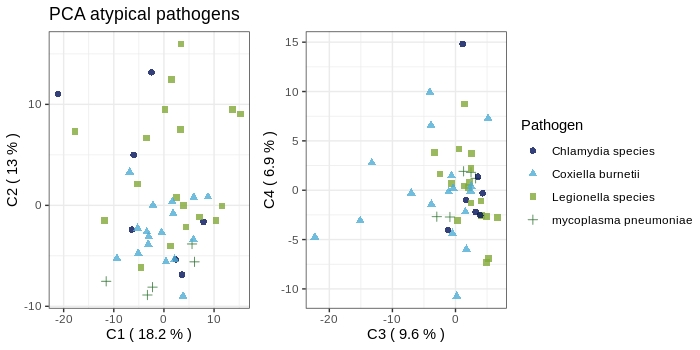


**S4 Fig. Principal component analysis (PCA) of the atypical pathogen group (log-transformed and standardized data) shows that there is no clear subgroup within the atypical group that would prominently drive the separation from the S. pneumoniae and viral infections.**
