# Supplementary material for: Metabolomic profiling of microbial disease etiology in community-acquired pneumonia
Source: PLoS One. 2021 Jun 4;16(6):e0252378. doi: 10.1371/journal.pone.0252378 (PMC8177549; doi:10.1371/journal.pone.0252378)
Supplement: S1 Table — (DOCX) [file pone.0252378.s006.docx]

**S1 Table. Summary of previous studies focusing on bacterial and viral respiratory tract infections and related metabolites.**

| **Compared groups** | **Matrix** | **Analytical method** | **Significantly altered metabolites**  **Upregulated Downregulated** | | **Reference** |
| --- | --- | --- | --- | --- | --- |
| 30 CAP vs 30 HC | Plasma | NMR | 1-methylhistidine | lactate, ketone bodies | Zhou et al. (2015) [12] |
| 30 CAP vs 38 TB | Plasma | NMR | lactate, pyruvate, lipids, ketone bodies | amino acids[leucine, isoleucine, valine], 1-methylhistidine, glucose, nicotinate, GPC | Zhou et al. (2015) [12] |
| 11 pneumonia vs  11 HC (children) | Plasma | UPLC-TOF-MS | uric acid, hypoxanthine, glutamic acid | L-tryptophan, adenosine-diphosphate | Laiakis et al. (2010) [13] |
| 11 pneumonia vs  11 HC (children) | Urine | UPLC-TOF-MS | uric acid, L-histidine |  | Laiakis et al. (2010) [13] |
| 47 pneumonia vs  47 HC | Urine | NMR | glucose, lactate, ketone bodies, amino acids [alanine, asparagine, isoleucine, leucine, lysine, serine, threonine, tryptophan, tyrosine, valine], carnitine, acetylcarnitine, hypoxanthine, fucose, myo-inositol, taurine, quinolinate, adipate, dimethylamine, creatine, 2-oxoglutarate, fumarate | citrate, trigonelline, 1-methylnicotinamide, succinate, levoglucosan, 1-methylhistidine | Slupsky et al. (2009) [14] |
| 30 CAP vs 46 TB | Plasma | UPLC-QTOF-MS |  | 12(R)-hydroxyeicosatetraenoic acid, ceramide (d18:1/16:0), cholesterol sulfate, 4a-formyl-4b-methyl-5a-cholesta-8-en-3b-ol | Lau et al. (2015) [15] |
| 42 Influenza A vs  30 Bacterial CAP | Plasma | NMR, GC-MS | 3-Methyl-2-Isovalerate, 3-Methyl-2-oxovalerate, 4-Hydroxybutyrate, Adipate, Alanine, Arabinonic acid, Asparagine, Aspartic Acid, Citrate, Citric acid, Fumerate, Histidine, Lysine, Methionine, Myoinositol, Phenylalanine, Serine, Threonic Acid, Threonine, Tyrosine, Uric acid, Urea | 2-amino Butanoic acid, Acetoacetate, Alkane, Benzoic acid, Beta-alanine, Carnitine, Dimethylamine, Formate, Glycine, Gulonic acid, Hexanoic acid, Leucine, Lactic acid, Pentadecane, Pyruvic acid, Quinic acid | Banoei et al. (2017) [17] |
| 55 RSV vs  24bBacterial pneumonia  vs 37 HC (children) | Urine | NMR | 3-Hydroxyisovalerate, 3-Indoxylsulfate, Acetoacetate, Betaine, Blue 1.06, Ethanolamine, Glutamate, N,N-Dimethylglycine, Pantothenate, Succinate, Tartrate, Uracil | Hippurate, Serine, Threonine | Adamko et al. (2016) [18] |

*Abbreviations: CAP: community-acquired pneumonia; VAP: ventilator-associated pneumonia; HAP: hospital-acquired pneumonia; TB: tuberculosis; RSV: respiratory syncytial virus; HC: healthy control; NMR: nuclear magnetic resonance; UPLC: ultra-performance liquid chromatography; GC: gas chromatography; TOF: time-of-flight; QTOF: quadrupole time-of-flight; MS: mass spectrometry.*
