# Supplementary material for: Metabolomic profiling of microbial disease etiology in community-acquired pneumonia
Source: PLoS One. 2021 Jun 4;16(6):e0252378. doi: 10.1371/journal.pone.0252378 (PMC8177549; doi:10.1371/journal.pone.0252378)
Supplement: S2 Table — (DOCX) [file pone.0252378.s007.docx]

**S2 Table. Additional patient characteristics per pathogen group.**

|  | ***S. pneumoniae* (N=48)** | **Atypical (N=47)** | **Viral (N=30)** |  |  |  |  | **P-value** |  |
| --- | --- | --- | --- | --- | --- | --- | --- | --- | --- |
| **Race** |  |  |  |  |  |  |  |  |  |
| Other | 1 (2.1%) | 1 (2.1%) | 0 (0%) |  |  |  |  |  |  |
| White | 31 (64.6%) | 46 (97.9%) | 30 (100%) |  |  |  |  | 0.81 |  |
| Missing | 16 (33.3%) | 0 (0%) | 0 (0%) |  |  |  |  |  |  |
| **Nursing home resident** |  |  |  |  |  |  |  |  |  |
| No | 46 (95.8%) | 47 (100%) | 25 (83.3%) |  |  |  |  |  |  |
| Yes | 1 (2.1%) | 0 (0%) | 4 (13.3%) |  |  |  |  | 0.07 |  |
| Missing | 1 (2.1%) | 0 (0%) | 1 (3.3%) |  |  |  |  |  |  |
| **Altered mental status** |  |  |  |  |  |  |  |  |  |
| No | 43 (89.6%) | 42 (89.4%) | 27 (90.0%) |  |  |  |  |  |  |
| Yes | 3 (6.2%) | 5 (10.6%) | 3 (10.0%) |  |  |  |  | 0.85 |  |
| Missing | 2 (4.2%) | 0 (0%) | 0 (0%) |  |  |  |  |  |  |
| **Respiratory rate** |  |  |  |  |  |  |  |  |  |
| Mean (SD) | 25.3 (6.64) | 25.5 (6.44) | 26.9 (7.32) |  |  |  |  |  |  |
| Median [Min, Max] | 25.5 [12.0, 40.0] | 26.0 [14.0, 40.0] | 29.0 [12.0, 44.0] |  |  |  |  | 0.81 |  |
| Missing | 8 (16.7%) | 8 (17.0%) | 6 (20.0%) |  |  |  |  |  |  |
| **Systolic blood pressure** |  |  |  |  |  |  |  |  |  |
| Mean (SD) | 131 (25.3) | 133 (15.8) | 137 (23.2) |  |  |  |  |  |  |
| Median [Min, Max] | 130 [88.0, 226] | 130 [99.0, 161] | 135 [90.0, 186] |  |  |  |  | 0.81 |  |
| Missing | 1 (2.1%) | 0 (0%) | 1 (3.3%) |  |  |  |  |  |  |
| **temperature** |  |  |  |  |  |  |  |  |  |
| Mean (SD) | 23.5 (8.41) | 24.2 (11.5) | 19.9 (9.40) |  |  |  |  |  |  |
| Median [Min, Max] | 24.0 [6.00, 42.0] | 24.0 [1.00, 41.0] | 20.0 [3.00, 39.0] |  |  |  |  | 0.37 |  |
| Missing | 1 (2.1%) | 0 (0%) | 0 (0%) |  |  |  |  |  |  |
| **pulse** |  |  |  |  |  |  |  |  |  |
| Mean (SD) | 104 (21.6) | 96.6 (18.6) | 94.3 (17.9) |  |  |  |  |  |  |
| Median [Min, Max] | 109 [60.0, 144] | 93.0 [50.0, 140] | 96.0 [60.0, 120] |  |  |  |  | 0.28 |  |
| Missing | 1 (2.1%) | 0 (0%) | 0 (0%) |  |  |  |  |  |  |
| **pH** |  |  |  |  |  |  |  |  |  |
| Mean (SD) | 12.7 (4.64) | 14.5 (4.28) | 12.0 (4.66) |  |  |  |  |  |  |
| Median [Min, Max] | 14.0 [3.00, 21.0] | 14.0 [3.00, 22.0] | 13.0 [1.00, 19.0] |  |  |  |  | 0.31 |  |
| Missing | 8 (16.7%) | 19 (40.4%) | 5 (16.7%) |  |  |  |  |  |  |
| **BUN** |  |  |  |  |  |  |  |  |  |
| Mean (SD) | 38.9 (24.9) | 46.0 (21.6) | 46.5 (24.1) |  |  |  |  |  |  |
| Median [Min, Max] | 35.0 [2.00, 81.0] | 48.0 [1.00, 84.0] | 52.0 [4.00, 82.0] |  |  |  |  | 0.50 |  |
| Missing | 1 (2.1%) | 0 (0%) | 1 (3.3%) |  |  |  |  |  |  |
| **sodium** |  |  |  |  |  |  |  |  |  |
| Mean (SD) | 132 (4.68) | 131 (5.59) | 136 (5.03) |  |  |  |  |  |  |
| Median [Min, Max] | 132 [117, 141] | 132 [119, 141] | 136 [125, 152] |  |  |  |  | 0 |  |
| Missing | 1 (2.1%) | 0 (0%) | 0 (0%) |  |  |  |  |  |  |
| **glucose** |  |  |  |  |  |  |  |  |  |
| Mean (SD) | 34.6 (13.9) | 34.7 (14.0) | 36.9 (17.6) |  |  |  |  |  |  |
| Median [Min, Max] | 35.0 [1.00, 58.0] | 35.0 [5.00, 59.0] | 42.0 [2.00, 59.0] |  |  |  |  | 0.85 |  |
| Missing | 3 (6.2%) | 0 (0%) | 5 (16.7%) |  |  |  |  |  |  |
| **hematocrit** |  |  |  |  |  |  |  |  |  |
| Mean (SD) | 11.0 (4.29) | 11.5 (3.75) | 10.5 (3.75) |  |  |  |  |  |  |
| Median [Min, Max] | 11.0 [1.00, 19.0] | 12.0 [1.00, 17.0] | 11.5 [1.00, 16.0] |  |  |  |  | 0.81 |  |
| Missing | 2 (4.2%) | 0 (0%) | 2 (6.7%) |  |  |  |  |  |  |
| **Partial pressure of oxygen** |  |  |  |  |  |  |  |  |  |
| Mean (SD) | 30.6 (16.5) | 31.8 (17.7) | 30.1 (16.3) |  |  |  |  |  |  |
| Median [Min, Max] | 34.5 [2.00, 56.0] | 33.0 [1.00, 55.0] | 37.0 [1.00, 50.0] |  |  |  |  | 0.92 |  |
| Missing | 8 (16.7%) | 19 (40.4%) | 5 (16.7%) |  |  |  |  |  |  |
| **Pleural effusion on x ray** |  |  |  |  |  |  |  |  |  |
| No | 39 (81.2%) | 45 (95.7%) | 25 (83.3%) |  |  |  |  |  |  |
| Yes | 8 (16.7%) | 2 (4.3%) | 5 (16.7%) |  |  |  |  | 0.31 |  |
| Missing | 1 (2.1%) | 0 (0%) | 0 (0%) |  |  |  |  |  |  |

*Data are presented as number (%) or mean (SD). Abbreviations: BUN: blood urea nitrogen.*
