# Supplementary material for: Metabolomic profiling of microbial disease etiology in community-acquired pneumonia
Source: PLoS One. 2021 Jun 4;16(6):e0252378. doi: 10.1371/journal.pone.0252378 (PMC8177549; doi:10.1371/journal.pone.0252378)
Supplement: S3 Table — (DOCX) [file pone.0252378.s008.docx]

**S3 Table. Overview of the number of metabolites included in the metabolomics platforms, measured in the samples and included in the data analysis.**

| Measurement platform | Number of metabolites included in platform | Number of metabolites measured in samples | Number of metabolites included in data analysis |
| --- | --- | --- | --- |
| Amines | 74 | 55 | 55 |
| Acylcarnitines | 48 | 28 | 28 |
| Organic acids | 28 | 13 | 13 |
| Negative lipids | 30 | 16 | 16 |
| Signaling lipids | 231 | 113 | 91 |
| Positive lipids | 185 | 149 | 144 |
| Total | 596 | 374 | 347 |
