# Supplementary material for: Metabolomic profiling of microbial disease etiology in community-acquired pneumonia
Source: PLoS One. 2021 Jun 4;16(6):e0252378. doi: 10.1371/journal.pone.0252378 (PMC8177549; doi:10.1371/journal.pone.0252378)
Supplement: Attachment — Submitted filename: Response to Reviewers.docx [file pone.0252378.s012.docx]

Dear Dr. Singanayagam,

Hereby we would like to resubmit our revised manuscript entitled “Metabolomic profiling of microbial disease etiology in community-acquired pneumonia.”

We have addressed the remaining reviewer comments in this revised version, for which we provide a specific response below. In addition, we have made a number of minor textual changes to further improve readability of the Discussion section.

We hope that this manuscript is now acceptable for publication.

Sincerely,

on behalf of all co-authors,

Ilona den Hartog and Coen van Hasselt

**Reviewer #1:**

I probably missed this point. When were the serum samples taken? In the morning before food? Has this been standardized?

As for 1.) no standardization for sampling times and conditions was applied. Please add in the disscussion if this may limit some (say which ones!) of the conclusions of the study and why.

**Response:**

Since the samples were not collected specifically for metabolomics analysis, no standardization for sampling times and conditions was applied.

In the Methods section we stated the following (lines 85-86):

*“The samples were taken from CAP patients within 24 hours after hospital admission.”*

Furthermore, we have added to the discussion the following (lines 348 - 356):

*“Furthermore, no standardization of sampling times and conditions was applied, e.g., patients had not fasted before blood sampling, which may influence the metabolite patterns found. Since variations in sampling conditions were unknown, we were unable to consider these in our analyses. However, we expect that the impact of not standardizing and correcting for these factors is limited because the noise in metabolite levels introduced by these factors is expected to be random with regard to the pathogen groups compared in this study. A standardized sampling approach could improve the sensitivity of the models to detect predictive metabolites because some noise is reduced. However, the specificity of the models with respect to the prediction of specific pathogens would be unchanged, since no correlation with pathogen groups is likely.”*
